# Implementation lessons for school food policies and marketing restrictions in the Philippines: a qualitative policy analysis

**DOI:** 10.1186/s12992-017-0320-y

**Published:** 2018-01-23

**Authors:** Erica Reeve, Anne Marie Thow, Colin Bell, Katrin Engelhardt, Ella Cecilia Gamolo-Naliponguit, John Juliard Go, Gary Sacks

**Affiliations:** 1 Global Obesity Centre (GLOBE), World Health Organization Collaborating Centre for Obesity Prevention Centre for Population Health Research School of Health & Social Development, Faculty of Health, Melbourne, Victoria 3218 Australia; 2 Menzies Centre for Health Policy, The University of Sydney, Sydney, NSW 2006 Australia; 3 Formerly of the Division of NCD and Health through the Life-Course, The World Health Organization Regional Office for the Western Pacific, Manila, Philippines; 4 Bureau of Learner Support Services, Department of Education Central Office, Pasig, Philippines; 5 Philippines Country Office, Western Pacific Regional Office of the World Health Organization, Manila, Philippines

**Keywords:** Nutrition policy implementation, Food marketing, School food policy

## Abstract

**Background:**

The school environment can enhance children’s skills, knowledge and behaviours in relation to healthy eating. However, in many countries, unhealthy foods are commonly available in schools, and children can be exposed to aggressive marketing by the food industry. Taking the perspective of policymakers, this study aimed to identify barriers and enablers to effective school food policy development and implementation in the Philippines.

**Methods:**

In May 2016, semi-structured interviews were conducted with 21 policymakers and stakeholders involved in school food policymaking and implementation in the Philippines. The Health Policy Analysis Triangle was used to identify interview questions and to guide the thematic analysis. These included the political and socio-environmental context, strengths and limitations of existing policy content, roles and behaviours of actors, implementation processes, policy outcomes, and opportunities to improve policy coherence.

**Results:**

The Department of Education’s policy ‘Orders’ represented a relatively strong policy framework for the education sector of the Philippines. However, a lack of human and financial resources for implementation, planning, and policy enforcement limited the impact of the policy on the healthiness of school food provision. Ambiguity in policy wording allowed a wide interpretation of the foods eligible to be provided in schools, and led to difficulties in effective monitoring and enforcement. Food companies used existing relationships with schools to promote their brands and compromise the establishment of a stronger food policy agenda. We found a motivated group of actors engaging in policy-oriented learning and advocating for a stronger policy alternative so as to improve the school food environment.

**Conclusions:**

The adoption of policy mechanisms being used to promote healthy dietary practices in the school setting will be strengthened by more robust implementation planning processes, and resources to support implementation and enforcement. Policymakers should ensure policy language clearly and unequivocally promotes healthier food and beverage options. Steps should be taken to achieve policy coherence by ensuring the objectives of one agency or institution are not undermining that of any others. Where there is reliance on the private sector for school resources, safeguards should be established to protect against conflicts of interest.

## Background

Unhealthy diets are one of the main risk factors for disability and deaths globally [1, 2]. For many children, school-based food provision and sales account for a substantial proportion of their food intake. The school environment is critical in establishing skills, knowledge and behaviours around healthy foods and diets [3, 4]. Even in schools however, children and adolescents are increasingly targeted by the food industry through aggressive marketing tactics, with evidence showing that food marketing towards people under 18 is dominated by unhealthy foods and beverages [5–7]. This is occurring in an environment where private-sector partnerships have emerged as an important, yet controversial [8], mechanism for subsidising the costs to government of education delivery, especially in low-resource settings [9].

Global recommendations for non-communicable disease (NCD) prevention, including the Global Action Plan for NCDs [10] and the Rome Declaration on Nutrition and Framework for Action (2015) [11], have consistently called for heightened action on nutrition in school settings. The World Health Organization’s (WHO) ‘Report of the commission on ending childhood obesity’ *(*2016) recommends establishing standards for meal provision to meet nutrition guidelines, eliminating unhealthy foods from the school environment and establishing mechanisms to safeguard public health from conflicts of interest [11]. School -based interventions promoting consumption of healthy food and non-alcoholic beverages are frequently reported to be among the most cost-effective diet-related approaches to NCD prevention [12, 13].

Despite these calls to action, implementation of recommended policies for healthy food provision in schools has been inconsistent and inadequate globally [14]. Evidence from high income countries, including Australia and Canada, has reported large disparities between food policy-making and food policy implementation in the school setting [15–18]. While health and education objectives with respect to the school setting are generally compatible, educational development often takes precedence over health programs to achieve academic accountability [19].

The barriers to and enablers of successful implementation of school food policies from the national and subnational levels are not well understood in contexts similar to the Philippines [20, 21]. One study from Laos found that a lack of strategic vision, inadequate planning, limitations to human and financial capital and the absence of institutionalised monitoring systems hindered national school health policy implementation [22]. Apart from this study, few others have examined factors affecting school food policy implementation in low-resource settings. The International Food Policy Research Institute’s 2016 *Global Food Policy Report* called for researchers to explore the technical, political and economic enablers and barriers to implementation and enforcement of nutrition initiatives [20]. Generating insight into the factors affecting sustained policy implementation within a given socio-political context is a useful step towards improving policy design and adherence [23].

Policy setting: the Philippines.

The Philippines is a highly urbanised, lower-middle income country in East Asia with high rates of poverty and unemployment [24]. The Philippines faces an extreme double-burden of malnutrition [25]: stunting affects over 30% of children under the age of 5 [26], and diet-related NCDs are steadily increasing. NCDs accounted for 67% of total deaths in 2014, up from 61% in 2010 [27]. Rapid economic growth, urbanisation and globalisation have had a significant impact on the food supply in the Philippines, where an array of ultra-processed foods and drinks are readily available at low cost [24, 28, 29]. Consumption of snacks, carbonated beverages and away-from-home meals is comparably high in the Philippines, with reference to other countries in the Region [30–32].

There are over 46,000 schools in the Philippines, providing kindergarten to Grade 12 education. In many densely-populated areas classes are delivered across two sessions per day, with sessions starting from 6 am and closing up to 6 pm. Education as a sector is centralised and organised by central, regional and divisional policy layers, with health and nutrition officer functions framed as a ‘learner support’ services. Department of Education Orders “Dep Ed Orders” are the overarching policy framework governing the rights, obligations and operations of the school setting, including the provision, marketing and sale of food.

While the country is currently without a national regulatory mandate to protect children from harmful marketing of unhealthy foods and beverages there is an industry-initiated self-regulated pledge called the *Responsible Advertising to Children Initiative* (the ‘Philippines Pledge’) [33].

This study aimed to understand barriers and enablers to effective school food policy development and implementation in the Philippines. It also sought to identify opportunities to develop more comprehensive policy frameworks in the area.

## Methods

We used qualitative policy analysis research methods to interview health and education officials in the Philippines and review relevant documentation related to barriers and enablers to realising policy outcomes [34, 35]. We used policy theory to inform all aspects of the study, including the development of the interview schedule and thematic analysis of the data collected. This encouraged inquiry beyond the level of description to examine the complex set of circumstances in which the policies were being realised [23]. Walt and Gilson’s ‘Health Policy Analysis Triangle’ was selected as the primary theoretical lens, as it was deemed to incorporate the key aspects that were likely to be relevant to this analysis, and has previously been used in similar contexts [22] [36]. The policy analysis triangle prompted investigation into multiple aspects of the policy process, recognising that the behaviour of actors, the process of policy-making and implementation, as well as the context in which policies are developed and disseminated all influence policy content and outcomes [34, 37].

### Study design and participants

ER conducted 21 semi-structured interviews with key informants across the Greater Manila Area in May 2016. The National Nutrition Centre selected the initial sample of informants, based on participants’ knowledge of the school food policy-making process and/or their role in school food policy implementation. Representatives from the National Nutrition Centre requested participation from interviewees with letters and follow-up phone calls. Other relevant participants were identified through snowball sampling. Eighteen interviews were conducted in English. Three food providers were unable to communicate in English, and so a translator was used in this case.

The participant sample included national-level policymakers from health, education and agriculture (9), municipality-level health and education officers (3), school principals (4), food providers (3), a senior nutrition researcher and a representative of the food regulatory authority. Interviews were recorded and handwritten notes were taken when interviewees preferred that the interview was not recorded.

Based on the components of the Health Policy analysis triangle, semi-structured interview questions centred on:

- the content of policy initiatives (content);
- governance of the school and food environments (context);
- implementation, communication, monitoring and enforcement mechanisms; and capacity support, tools and resources (process);
- stakeholder knowledge of key policies, and their views on the effectiveness of current school food regulations (outcomes); and
- the perspectives, roles and influence of actors on implementation and outcomes (actors).

The content of the semi-structured interview schedule was adapted to the role and expertise of the interviewees, and also updated iteratively to pursue relevant information identified through the interviews [38].

### Document and webpage review

To inform the analysis of policy context and supplement the interview data, a literature search was conducted to identify documents describing and regulating school food environments in the Philippines. We reviewed policy documents provided by informants, and performed an electronic search through 20 years of Philippines Department of Education policy ‘Orders’ to search for directives relating to food and nutrition.

We also conducted a content analysis of global food company websites to identify activities being delivered by food companies in the education setting of the Philippines. The websites in this content analysis were selected as those companies listed on an Adopt-a-School Program (Stanfilco, Coca-Cola Far East Limited, Coca-Cola Foundation Inc., Pilmico Foods, Uniliver), and nine companies listed as signatories on the Philippines Pledge (including Unilever, Nestle, Coca Cola, Mars and Pepsico) [33].

In May 2017, ER visited websites for each company to view evidence of philanthropic work in schools. Where images depicted visible branding in schools (confirmed through accompanying text), these were captured using a software add-on called FireShot Capture [39].

### Analysis

Data analysis used the key components of the health policy analysis triangle as a foundation, supplemented by concepts drawn from other policy theories including the advocacy coalition framework [40, 41], and the multiple streams theory [42]. Analysis focussed on: strengths and limitations of policy content, roles and behaviours of actors, the political and socio-environmental context, opportunities to improve policy coherence, and characteristics of implementation. We also examined policy outcomes based on stakeholder interpretation and perspectives on the policy. ‘Policy outcomes’ were added as a construct to our analytical framework because outcomes are a proxy indicator of successful implementation, an approach applied by other researchers undertaking similar case study research [36].

The structure and content of the current food policy mechanisms were analysed with reference to the Philippines Food-Based Dietary Guidelines and the Food Pyramid for Filipino children and teens, and WHO’s Set of Recommendations on marketing food and non-alcoholic beverages to children [43].

We structured the results according to the key concepts in the analytical framework (policy context, policy contents, policy process and policy outcomes), whilst drawing out barriers and enablers of policy implementation. We presented the discussion according to the key themes that emerged from the study, with a focus on enhancing generalizability of the findings to other settings and promoting policy learning [44, 45].

### Ethical approval

This study received ethical approval from both the Human Ethics Advisory Group at Deakin’s Faculty of Health (approval HEAG-H47/2016) and the Philippines National Ethics Committee with the Philippines Council for Health Research and Development (NEC Code 2016–002-Reeve-Healthy Food and Beverages).

## Results

### Policy context

#### Nutrition governance

Food and nutrition policies are generally developed under the mandate of the National Nutrition Council, a high-level multisectoral body responsible for the development, implementation and monitoring of food and nutrition policies, including the Philippines Plan of Action on Nutrition (2011–16).

The Philippines Government has decentralised most governance responsibilities to approximately 1490 local government units and municipalities across 81 provinces. At the base of governance arrangements are small village-like administrative zones called ‘Barangays’. To improve community reach in implementing the Plan of Action on Nutrition, a voluntary network of over 19,000 community based nutrition workers called ‘Barangay Nutrition Scholars’ are trained and supervised to undertake nutrition assessment and counselling in communities across the country [46]. Some interviewees noted that these Nutrition Scholars could, in theory, be given greater responsibility for promoting school food regulations, however this would be in direct competition with other priorities.

*Who will be the one to enforce that ban [on the sale of sugar-sweetened beverages and/or other unhealthy foods and beverages]? There could be the sanitary inspectors or barangay health workers … or community health workers, barangay nutrition scholars, but then that would add to their roles…They have so many functions to attend to already.*

*Interview 30, Nutrition Official.*

*But if you’d look at the data, the number of under nourished children is still higher compared to the overweight ones. So, in terms of a priority I think this is not much of a priority [for Barangays].*

*Interview 24, Nutrition Official.*

#### School food provision

According to informants at the Department of Education, ‘most’ schools have a canteen or store, and ‘most’ offer a school feeding program to the most vulnerable (‘at-risk’) children. Many children also bring food to school, reportedly dominated by highly processed products (including hot dogs and fruit juice). Public school canteens and stores are most often run by canteen workers, parents or other volunteers, and in some cases by teacher’s cooperatives. In the latter scenario, the income of teachers is directly impacted by canteen profitability.

All interviewed policy officials and principals were committed to deliver school feeding programs for undernourished children. According to the policy, 35% of all profits generated from the canteen are directed towards the school feeding program for undernourished children [47]. For some schools, the need to fund the feeding program created a conflict for enacting tighter restrictions on the sale of processed packaged foods.

#### Availability of affordable processed foods around schools

The high density of stalls selling unhealthy foods around the school perimeter was noted as a major challenge to ensuring children consume healthy food during school hours. School workers and policy makers both noted the influence of ‘sari-sari’ stores (small convenience stores) on children’s exposure and consumption of unhealthy foods. One stallholder outside a school described that school children most commonly purchased biscuits, sugar-sweetened powder mixes for drinks, juice, cola, fudgy bars and biscuits.

*Small sari-sari stores, that is the big problem, mostly, in the public schools because they know that children really love those junk foods….*

*Interviewee 29, Policy maker.*

Many interviewees felt it would be difficult to put in place restrictions on ‘sari-sari’ store sellers due to the potentially negative impact on sellers’ livelihoods, and that it would be difficult to ‘police’ food sellers.

Affordability was also cited as having a significant baring on children’s purchasing decisions in and around the school environment, with interview participants reporting that highly-processed foods (e.g. extruded snacks) were “cheap” compared with healthy locally prepared alternatives (steamed corn cob, fruit or local peanuts).

*In terms of the affordability, well, it’s still a big factor because if you buy the chips, you can spend only two pesos [USD 0.04]. Unlike if you buy fresh fruit, it could be five times or as much, 10 pesos [USD 0.2].*

*Interview 30, Education Official.*

#### Food company philanthropy and promotion in Filipino schools

The Philippines Department of Education instituted the ‘Adopt-a-School’ Program in order address resource shortages to the education sector [48, 49]. The program aims to improve education quality and opportunities by engaging private partners to provide infrastructure, funding and other resources in exchange for tax incentives [49, 50]. Partners listed by the Department of Education include the Coca-Cola Foundation, San Miguel and Stanfilco [48] – all large food companies operating in the Philippines.

*And we understand that there are some conflicts of interest within the school system…school equipment and some of the materials needed in school are lacking. So, the school actually resort[s] to some fundraising and partnership.*

*Interview 22, Nutrition Official.*

Companies that partner with the government as part of the Adopt-a-School Program are reportedly not allowed to advertise their products in schools, but guidelines outlining criteria for participation in the program do not specifically reference restrictions on brand visibility or marketing in schools [49].

*There is an engagement with partners through the Adopt-A-School Program…you are not allowed to advertise but you can promote the program. So, if there is a partnership, you can see the logo…but they promote the program, not their product.*

*Interview 21, Education Official.*

Policymakers were concerned that the Adopt-a-School Program might provide a barrier to imposing restrictions on marketing with schools.

*I think that’s [regulating marketing in schools] really…difficult because we have this Adopt-a-School Program. We encourage companies to partner with us in adopting a school, maybe they provide a school building in exchange of tax incentive. So, for example, the Coca-Cola Foundation – they are donating classrooms in exchange of tax incentives.*

*Interview 20, Education Official.*

### Policy contents

#### Food provision in schools

At the time of these interviews, Department of Education Order Number 8 (2007) was recognised by all relevant interviewees as the prevailing policy mechanism governing food provision and sales in the school setting. The Order stated that foods provided must be “nutrient rich”, then lists “*root crops, noodles, rice and corn products in native preparation”*, fruit and vegetables, and “*fortified food products labelled rich in protein energy, vitamins and minerals”.* It prohibits the sale of carbonated drinks and “sugar-based synthetically or artificially flavoured juices” and “junk foods” (defined as “*foods which may be detrimental to children’s health”)*. However, it does not specify the frequency that specific foods should be provided and it does not have nutrient-based or food-based criteria to classify foods.

The foods explicitly promoted in Department of Education Order Number 8 (2007) are consistent with the Government’s Philippines Food-based Dietary Guidelines (2012), but they do not promote the full range of food groups recommended by the Guidelines, for instance fish, lean meat, poultry, eggs, dried beans, nuts, milks, and fish. The Order does not specifically restrict intake from salty, fried, fatty and sugar-rich foods, as recommended by the Philippines Food-based Dietary Guidelines (2012).

All products bearing the words ‘Sangkap Pinoy’ are allowed under the Order number 8 (2007). This is a government ‘seal’ signifying the fortification of a product with nutrients as per Republic Act 8974 [51]. Interviewees indicated that manufacturers have capitalised on this seal by fortifying packaged products including extruded snacks, sweetened biscuits, hot dogs and sugar-sweetened beverages. All Sangkap-approved foods can be provided in schools.

*We do not sell junk-food, only those foods with Sangkap Pinoy which are acceptable for kids.*

Interview 14, Principal.

*Currently, the Department Order includes also fortified foods, but these fortified foods are mostly chips. They may be high in vitamin A, but the rest is just calories-.*

Interviewee 30, Health Official.

#### Restrictions on unhealthy food marketing in and around schools

At the time of these interviews, there were no national marketing controls in the Philippines. Within the school setting, another Department of Education policy, Order 37 (2010), is a generic marketing control that prohibits “the use and or display of school signage showing commercial advertisements, sponsorships and endorsements”.

The ‘Philippines Pledge’ lists global food subsidiaries including companies Coca-Cola Far East Ltd, Mars Philippines, Kraft Foods Philippines Inc, Nestle Philippines Inc, Unilever Philippines Inc and Pepsico International (2010) as signatories, and pledges not to provide any commercial material to schools unless “agreed with school administration for educational or information purpose”. The pledge commits to restricting advertising of products that do not fulfil “specific nutrition criteria…based on accepted scientific evidence and/or applicable national and international guidelines relevant to children” [33]. There is no mention of which nutrient criteria or international guidelines underpin this pledge.

### Policy processes

#### Agenda setting and policy development

Three key issues emerged as barriers to adopting comprehensive healthy school food provision and marketing policies. In the first, interviewees reported that policymakers who had tried to bring attention to the contribution of food provision and marketing to unhealthy eating habits were faced with requests from politicians for stronger evidence of the linkage between sugar consumption and poor health. For example, a House Bill to tax sugar sweetened beverages, introduced for the first time in 1999 had been opposed because policymakers could not provide evidence that sugary beverages posed a “social cost” [29]. Additionally, multiple interviewees indicated that political leaders would refuse to acknowledge a problem with food supply and consumption if research was not underpinned by a Filipino dataset.

*Well, actually there are a lot of studies but it’s international. And our legislator would say, “Oh, but that’s international, we don’t have local evidence.”*

*Interview 22, Nutrition Official.*

Interviewees indicated that the types of evidence called for included ‘scientific data’ proving unhealthy foods should not be provided to children; data on local consumption patterns among children within and outside school; clarity around which foods exceed levels of sugar deemed detrimental to Filipino children; clinical evidence of the impact of childhood obesity in the Philippines, linkages between sugar intake, obesity and health outcomes; and cost-benefit analyses of marketing restrictions. They also called for nutrient profiling systems to be developed using local data.

The second barrier identified by interviewees was the widespread concern amongst policy makers that restrictions would interrupt the profitability and growth of food companies. The country draws significant income from primary products (like rice and sugar), and food manufacturers contribute to income, employment, economic growth.

*Yeah, it’s very difficult to say that the product is unhealthy. It may kill the business industry. It’s like saying you should not buy their product, to stop doing business.*

*Interview 17, Food Regulations Officer.*

Thirdly, interviewees in the health and education arms of government indicated that they anticipated that the food industry would vigorously oppose any efforts to reduce marketing or restrict the sale of unhealthy foods in and outside of schools, and that it would have a strong influence on political commitment to this agenda.

*We expect more opposition coming from the food manufacturers…We don’t know how they can influence our legislators. Just the way when an amendment to the milk code was being proposed, there was such a strong lobby among the legislators.*

*Interview 30, Health Official.*

*Food companies are big companies, you know, they have lots of money...we will not expect them just sitting down, they will do something to counter our efforts.*

*Interview 24, Nutrition Official.*

Informants cited examples of food companies deliberately obstructing the public health agenda by coercing policymakers and engaging in political advocacy against health legislation. Policy makers at both national and local government levels reported being directly approached by food industry executives and pressured to lift policy initiatives aimed at improving food in schools.

*The Pepsi and Coca Cola they work together, can you imagine that? They came to the [Dep Ed] office, Pepsi, Coca Cola…They are the Beverage Association of the Philippines.*

*Interview 23, Education Official.*

*There’s a very strong lobby of the soft drinks industry. Already I learned that the association of food, beverages are already asking the Dep Ed to lift that ban [on sugar-sweetened drinks in schools]. The same way that there was a proposed … There was a bill on increasing the tax for soft drinks. There was a lot of opposition in that. We expect more opposition coming from the food manufacturers.*

*Interview 30, Health Official.*

#### Implementation

At the national level, there was no evidence of implementation planning for the execution of (Department of Education Order Number 8 (2007), no resources specifically dedicated to implementation, and limited capacity building opportunities to imbed and sustain policy adherence. Most implementing actors (schools, local government education divisions, local government health departments, schools, principals and teachers) reported they had been trying to interpret the policy without adequate tools, training or support to guide them. There was a call for clearer guidance and capacity building opportunities to better prepare school workers to provide healthier foods and beverages.

*Schools are always asking for information on what is healthy and not healthy? Can you provide us a list of foods that are healthy and unhealthy? We asked our Division Health Department for a list of such foods but they did not have one.*

*Interview 18, Division Education Officer.*

*Well this one [Dep Ed Order Number 8] is – is not you know, it’s not – cause it’s not very clear…this is subject to adaption by the schools. So, I don’t think this a strong enough tool to be used.*

*Interview 24, Nutrition Official.*

The motivation and capacity of the principals to promote Dep Ed Order Number 8 was viewed as crucial for sustaining implementation. It was noted by several informants that principals were overwhelmed by the sheer number of ‘priorities’ handed down to them, compromising their ability to govern school food policies.

*If you’re in the school there’s so many policies that goes down but there’s only one person that’s supposed to do it, it’s the principal. So, if prioritisation is [to happen, it needs be] from the principal. And I feel that the school has a lot of needs yet for curriculum, for infrastructures, health will be third of the priority or at least last. So in monitoring of how the school canteen will be managed will be their least priority. They will always prioritise curriculum, content, teacher capacity first.*

*Interview 23, Education Official.*

Amongst school principals interviewed, there were diverse views on the importance of nutrition relative to other considerations. For example, one principal had gone to great lengths to promote a healthy food environment in the school, with initiatives including a healthy school canteen, nutrition education, and a large ‘Gulayan sa Paaralan’ (vegetable program) to feed undernourished children. He had also built a garden wall around fences to prevent vendors selling ‘junk’ food through the fence to the children.

*Most of the pupils go to the fence of the school and the ambulant [mobile] vendors just at the other side of the fence and they exchange goods…So, what I did is I put a barrier, one metre apart [from the fence]…and then I made it a vertical [vegetable] garden.*

*Interview 14, School Principal.*

#### Monitoring and enforcement

Department of Education Order Number 8 (2007) delineates responsibility for monitoring and enforcement to Divisional and Regional Offices of the education department. However, other government agencies were also being engaged in school food monitoring. Interviewees identified that a lack of adequate monitoring and enforcement was a key factor impairing the effectiveness of the policy. This related to a lack of human and financial resources at the national, subnational and local government levels [47], the lack of clarity around what criteria to use for monitoring, and the absence of tools to aid assessment, reporting and enforcement.

*Number one [challenge] is who’s going to do the monitoring? Because it’s another agency, so it’s Dep Ed and here is the health department monitoring the Dep Ed.*

*Interview 12, Local Government Official.*

*What will be the tool that should be used during the monitoring? For us, we just check. And then we look at, you know, even the hygiene of those who are preparing the foods in the canteens.*

*Interview 12, Local Government Official.*

The 2007 policy does not list the nutritional quality of food among its other reporting requirements. While the Order provides a grievance mechanism and introduces sanctions for violations, in the case of “simple violations”, a warning is provided to principals. When questioned about enforcement of school food policies, no stakeholder raised the possibility of sanctions, and there was an inference amongst interviewees that the Orders would only be enforceable (and thus adhered to) should they become legislated.

*There are no sanctions actually aligned with the policies.*

*Interview 21, Education Official.*

*I know that they have issued this order, but they have not really monitored the implementation of this. There’s no conscious effort, really, to monitor the implementation. But if it is a law…they have an oversight function. They can actually investigate if it is really being implemented and they can require reports from agencies on how they have implemented these things.*

*Interview 22, Nutrition Official.*

### Policy outcomes

#### Effectiveness in promoting healthy consumption at school

There were no routine measures on which to gauge the effectiveness of the policies in place, however most interviewees alluded to the fact that existing policy mechanisms were not sufficiently protecting children from being exposed to the marketing and sale of unhealthy foods and drinks in and around schools.

*In general, Dep Ed orders are not very definite …implementation’s also a problem even though there’s a ban [on sale of sugar-sweetened beverages]. We have banned carbonated drinks but if we go to the school -why are you still (selling) Coca Cola?*

*Interview 23, Education Official.*

*Dep Ed has a policy on sugary drink but they’re still selling sugary drink, I don’t know what happened...We have already done monitoring…We asked the school canteens the available foods like for example any processed food or biscuits. And we found out that’s all, you know, sugary, it’s all sweet.*

*Interview 12, Local Government Official.*

*We received some feedback that there are some violations on the Dep Ed order. Some of the parents…they still see some unhealthy foods in the canteens.*

*Interview 20, Education Official.*

Interviewees described commonly provided foods in schools as including bread, noodles, juice drinks, cupcakes, biscuits, fruits, packaged snacks and sweetened rice. One Division-level education official spoke of promoting white bread, homemade cakes and biscuits as healthy foods because they were allowable under the Orders. Fortified versions of sodas and sweetened juices, sweet and savoury biscuits, chocolate malt, chips and extruded snacks baring the Sangkap seal were also being provided. There was also variability in the nutrition literacy amongst the principals interviewed.

*Biscuits are good, but you know it’s the spread [that can be problematic], but if it’s a plain biscuit, probably it’s good. It’s a healthy food.*

*Interview 12, Local Government Official.*

*I allowed Nestlé and Milo and milk because in my own opinion, using my own criteria, they are healthy for me. I don’t know if they’ll pass in this [nutrition] criteria.*

*Interview 14, School Principal.*

According to the Department of Education, some schools have pursued unhealthy food bans under their own initiatives, and the degree of support for healthy food promotion is based on the perspective of school administrators with regard to the importance of healthy consumption to children and their development.

#### Presence of food branding in schools

Interviewees provided numerous examples of food company sponsorship and promotion in and around the school setting. These included ‘nutrition lectures’ provided to parents by Nestle, Jollibee scholarships, Milo sports days, Pepsi gymnasium floors, Coca-Cola red painted school buildings, and branded soft drink bottles used in school gardening projects. Two principals referred to the Department of Education Orders restricting commercial activity, but provided examples of commercial activity when prompted for examples of support and donations from private companies. One principal repeatedly referred to the Orders but proudly promoted his extensive cola-bottle vegetable garden.

*There are Coca Cola red schools, there are McDonalds little red high school libraries!*

*Interview 23, Education Official.*

*Usually Nestlé company conducts parents’ education on nutrition…[Nestle?]…Nestle, that’s right... they give fliers to parents. Here in school, but sometimes I can see that it’s very limited.*

*Interview 14, School Principal.*

*We partnered also with one school and they’re promoting healthy lifestyle so their gym, actually the flooring has Pepsi [logo] on it…and of course the refrigerators and everything, it’s either Coca Cola or Pepsi!*

*Interview 22, Education Official.*

#### Food branding evident through school support

A scan of websites belonging to the Philippines arm of selected food companies revealed that food companies support schools in the Philippines by providing feeding programs, scholarships, resources including textbooks and computers, through infrastructure and maintenance, and by offering scholarships, training and learner support.

**Fig. 1:**
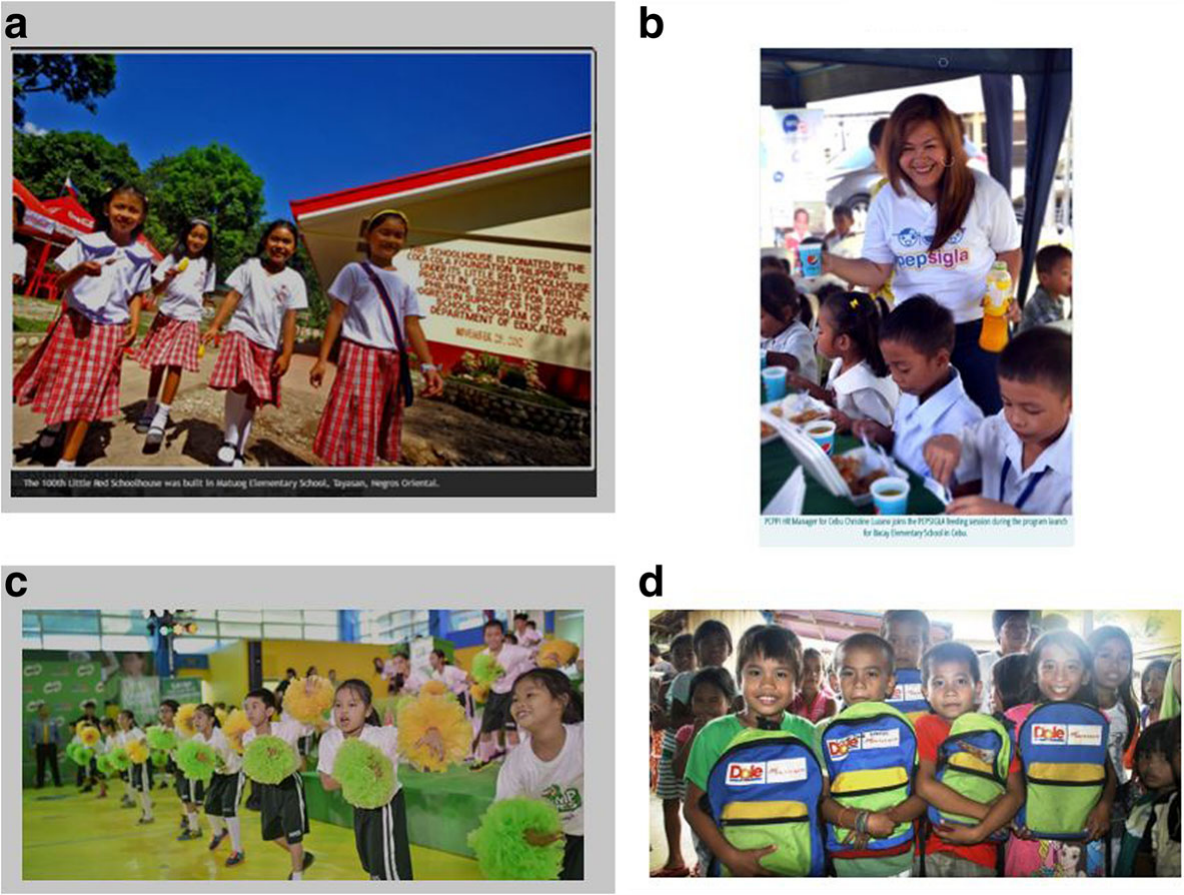
Food marketing in schools in the Philippines. Panel (**a**) Coca Cola Foundation schoolhouses. Panel (**b**) Pepsigla school feeding programs. Panel (**c**) Nestle’s Milo Champ Moves. Panel (**d**) Dole’s Adopt-A-School program

The scan also found numerous images depicting prominent company branding in school settings (Fig. 1). For instance, the Coca Cola Foundation site promotes infrastructure support provided by them to the Department of Education and features an image of a red schoolhouse, with the Coca Cola logo on its wall, and red Coca Cola logos on umbrellas in the background [52]. Pepsi promotes its ‘Pepsigla’ school feeding programs on its site. In images linked to the program, staff are wearing Pepsigla-branded t-shirts and serving a yellow drink to children in Pepsi-branded cups [53]. On Nestle’s site, their school-based program Milo Champ Moves is promoted by an image depicting children wearing Milo t-shirts, dancing in front of large Milo banners with green and gold pompoms [54]. Dole’s webpage depicts images featuring children holding Dole branded school bags to promote their participation in the Adopt-A-School program [55].

## Discussion

This study found that, despite a relatively strong policy mechanism for healthy school food provision and marketing (that includes a ban on the provision of sugar-sweetened drinks in schools, amongst other restrictions), the lack of human and financial resources for implementation, monitoring and policy enforcement restricted its impact. The study also found that clearer policy wording would likely improve both interpretation of the policy while better enabling accountability. Additionally, external actors, particularly from large food companies, were found to be compromising policy processes and agenda-setting. However, we did find a motivated group of policy-entrepreneurs advocating food policies to protect children.

This research identified several opportunities for strengthening implementation of healthy school food provision and marketing policies in the Philippines, outlined below. By considering the findings of this study through the lens of theories of policy learning [56], our discussion of findings focusses on key, globally-relevant dimensions of school food policy, which are likely to be relevant for informing the development of the design, governance and implementation of policies in other contexts. We have identified below key opportunities to strengthen policy design and implementation, including: specific and effective policy design; early consideration of implementation; identification of policy synergies; and management of conflicts of interest.

### Strong, unambiguous language

The healthy school food provision policies analysed here enabled a broad interpretation of their contents, demonstrating the need for countries to address ambiguity in policy language and ensure they unequivocally promote healthier food and beverage options. The success of school food policies may, at least in part, be predicated on the way in which ‘healthy’ foods are identified, categorised and subsequently managed, because differences can result in significant inconsistencies in interpretation, ‘strictness’ [57–60], and subsequent accountability of a policy. Internationally, there are a range of systems being employed to classify the provision of food and beverages in the school setting [4, 61–65]. Policy makers need to consider the most appropriate system for their context, depending on the policy goals and implementation considerations.

In relation to the industry-led marketing controls, we found that the Philippines Pledge was a weak set of standards which lacked in transparency and accountability. This is consistent with voluntary food industry initiatives adopted elsewhere [8, 66–68], with evidence that they have not been effective in reducing children’s exposure to marketing in the Philippines [69], Australia [70], Canada [71, 72] and Spain [73].

### Emphasising implementation and accountability

The absence of goals, targets and implementation plans is a common limitation of nutrition policies globally [14], and poor implementation planning has been shown to negatively affect school policy uptake in other countries [22, 74]. The adoption of policy mechanisms being used to promote healthy dietary practices in the school setting would have been strengthened by more robust implementation planning processes, with a communication plan, clear delineation of responsibilities for monitoring and enforcement, tools and resources to support implementation, and dedicated funding for capacity development. Adherence to school food policies is improved when investments have been made in the knowledge and capacities of school food workers [22, 74, 75] and through the provision of appropriate information and implementation support [75, 76]. Identification and investment in the human and financial resources required to fully imbed and enforce the policy is thus clearly an important factor [15, 77].

The food environment external to the school provided easy access to non-policy compliant foods. Local level factors have similarly been found to compromise school nutrition policy implementation in other settings, including the proximity of stores selling unhealthy alternatives and the prohibitive costs associated with policy-compliant foods [78]. Ongoing communication and consultation between policymakers and stakeholders has been effective in managing these (and other) factors, as well as enhancing the sustained compliance to school nutrition policies [78–80].

Weak accountability mechanisms further limited the success of policies examined here. Routine monitoring and accountability mechanisms are reportedly critical to the successful adoption and maintenance of school food policies [15, 22, 77, 81]. In lower-resource settings, there is generally reduced capacity for the implementation, enforcement and regulation of health policies [34], necessitating a greater degree of innovation in order to overcome such limitations. In Brazil, a number of collaborating universities were engaged to support the implementation and monitoring of the Brazilian School Feeding Program [82]. In other countries, the priority afforded to food policies in the school setting has been elevated through school recognition and reward systems, the introduction of coveted capacity building opportunities [83], and through the integration of food policy indicators into school performance management processes [84]. In the context of a public service that has multiple competing demands and a relatively low-level of resources, the Philippines could explore opportunities to synergise food policy enforcement through cross-sectoral partnerships, for instance by pushing for engagement on this issue by the Philippines Public-Private Partnerships Centre.

### Identifying and managing conflicts of interest in school food policymaking

The Philippines Government is highly reliant on private partnerships to address resource challenges in the education sector [85]. The private sector is clearly contributing to improving quality, equity and opportunities in education in the Philippines. However, we found that food companies were using their engagement with schools to promote their brands, for example by delivering feeding programs or giving ‘nutrition talks’. Although the Philippines Department of Health is not allowed to receive financial or material inducements from manufacturers of breastmilk substitutes [86], there are not equivalent restrictions in the Education sector. For example, Nestle provides support to the education sector through a Milo sports program [54]. This is consistent with previous research in finding that voluntary and industry-led initiatives may not be effective in protecting children from marketing by food and beverage companies [67, 69].

Resources provided through industry partnership approaches such as the Adopt-a-School program create a potential conflict for policymakers and school workers seeking to implement healthy school food provision and marketing policies. For example, if the Department of Education was to enforce marketing restrictions in schools, it may be at the expense of infrastructure and other resources being provided to them by food companies. According to WHO, food company sponsorship and participation in schools presents a high risk for conflict of interest [11, 67]. In a life-course approach to nutrition, good nutrition during school-age would be considered as high a priority as it is during pregnancy, infancy and early childhood, and have the same policy protections afforded to it [87].

Low resource settings are likely more vulnerable to this conflict because there is an increased reliance on private finance, and the benefit from engagement is comparably high [67]. Strategies to reduce conflicts of interests in school food policy include improved identification of potential conflicts and then introducing partnership eligibility criteria [8, 67] and ethical conduct codes [8], reinforcing accountability mechanisms [8, 67] and adopting complimentary statutory recommendations [67]. If food companies cannot comply with marketing bans then divestment from food industry partnerships may be required.

### Policy learning within the Department of Education

After this research was undertaken in 2016, the Department of Education in the Philippines initiated a rapid process to develop and adopt a new Dep Ed Order to surpass Order Number 8. This process was led by officials who were interviewed as part of this study and actively participated in regional WHO-led meetings advocating similar policies, indicating that policy officials were engaging in policy-oriented learning [88]. The newer policy, Order no 13 (2017), contains a more extensive policy content, including a food classification system that has been aligned to national food guidelines (‘Pinggang Pinoy’) [89]. It also restricts marketing of unhealthy food and beverages in and around schools (specifying a 100 m radius), and provides a 3-month lead-in time for schools to remove food branding on equipment and infrastructure. The newer Order delineates some responsibilities for promotion, capacity building and monitoring duties across different governance levels, and is being supported through extensive consultations, training opportunities and with tools. This policy learning took place within a relatively short period of time, providing an example of how policy coherence can occur through the ‘coupling’ of problems with solutions by policy entrepreneurs in the right political climate [40]. The recent passing of House Bill 3365 imposing a tax on sugary drinks after 18 years of advocacy is a further example of persistence in policy development, and the importance of capitalising on appropriate policy windows [90].

An opportunity for stronger policy that still remains, is the adoption of regulations to comprehensively protect children from marketing of unhealthy food and beverages, which is a priority item on the legislative agenda of the country’s multi sectorial nutrition agency, the National Nutrition Council. This study provides evidence that the influence of the food industry presents a risk to the progress of this agenda in the Philippines, and contributes to the lack of policy action in this area. The co-opting of political leaders by food and tobacco companies, as well as other corporate political activity, has the potential to undermine health policy making [66]. Policy makers will need to be prepared with strong justifications to underpin policies if they are to counter the tactics of the food industry. Conditions for this will be more favourable if policy makers simultaneously engage the support from policy entrepreneurs, civil society and other institutions [88, 91].

### Strengths and limitations of the study

The key strength of this research was that it directly involved policymakers from multiple levels of governance in the Philippines, and drew on the in-depth insights and perspectives they offered. Due to the extensive and well-organised policy material of the Department of Education, we were able to triangulate key information about sector partnerships, operations and policy processes against government policy materials. The policy analysis frameworks used have delivered an enriched understanding of the key influences on policy effectiveness, based on established policy science theory.

Nevertheless, the study had several limitations. Only a small proportion of policy makers involved in this policy landscape were included in the study. Moreover, while National, Division and Municipal level informants were interviewed, we were not able to interview any Provincial-level policymakers. Thus, the insights expressed by participants may not reflect the full range of views from relevant decision-makers. However, where possible, information provided by informants was cross-checked against the extensive and well-organised policy archives maintained by the Department of Education.

Without standardised and collated monitoring systems there was no data available on school food provision. Therefore, assertions on outcomes were based on qualitative contributions from the small range of interview participants. This was supplemented by the review of food company websites, but this was limited in that it could not date the activity depicted in the images found.

## Conclusions

This study found that in the Philippines, the adoption of tight regulations to protect children from the provision and marketing of unhealthy foods and beverages in the school setting was limited by the lack of resources and capacities for implementation planning, support and enforcement, a vague delineation of actor responsibilities, and potential conflicts of interest with food companies. This was compounded by unclear policy parameters, and ambiguous categorisation of foods to be restricted or promoted. These findings indicate that countries should facilitate implementation planning processes during policy development to establish goals, targets and activities, and measures for accountability and resource allocation. They should also carefully consider how food guidelines and policies will be interpreted, promoted and monitored. Subsequent efforts by policy makers in the Philippines after the study was conducted, have addressed this somewhat through clearer delineation of duties and responsibilities across government, although the availability of resources may yet constrain effective policy implementation.

Countries and organisations disseminating their lessons on policy making through global networks promote transnational policy learning [56]. Specific findings from this research suggest that the establishment of an agenda to fully protect children from exposure to unhealthy food and beverages in the school setting is likely to require greater resources, identification and management of conflicts of interest, strong co-ordination by a coalition of policy entrepreneurs, civil society and other institutions, and processes to ensure policy coherence across government institutions. Additionally, the standards provided through international institutions such as WHO can promote a convergence of policy objectives, principals and norms [56].
